# Out-of-equilibrium gene expression fluctuations in the presence of extrinsic noise

**DOI:** 10.1088/1478-3975/acea4e

**Published:** 2023-08-10

**Authors:** Marta Biondo, Abhyudai Singh, Michele Caselle, Matteo Osella

**Affiliations:** 1 Department of Physics, University of Turin and INFN, via P. Giuria 1, I-10125 Turin, Italy; 2 Department of Electrical and Computer Engineering, Department of Biomedical Engineering, Department of Mathematical Sciences, Center for Bioinformatics and Computational Biology, University of Delaware, Newark, DE 19716, United States of America

**Keywords:** gene expression, stochastic models, noise

## Abstract

Cell-to-cell variability in protein concentrations is strongly affected by extrinsic noise, especially for highly expressed genes. Extrinsic noise can be due to fluctuations of several possible cellular factors connected to cell physiology and to the level of key enzymes in the expression process. However, how to identify the predominant sources of extrinsic noise in a biological system is still an open question. This work considers a general stochastic model of gene expression with extrinsic noise represented as fluctuations of the different model rates, and focuses on the out-of-equilibrium expression dynamics. Combining analytical calculations with stochastic simulations, we characterize how extrinsic noise shapes the protein variability during gene activation or inactivation, depending on the prevailing source of extrinsic variability, on its intensity and timescale. In particular, we show that qualitatively different noise profiles can be identified depending on which are the fluctuating parameters. This indicates an experimentally accessible way to pinpoint the dominant sources of extrinsic noise using time-coarse experiments.

## 1. Introduction

Cellular processes are subjected to stochastic fluctuations. These fluctuations (or noise) can lead to phenotypic differences even in genetically identical cells sharing the same history and environment.

Noise can often be detrimental for the cell since it affects the precision and reliability of several processes, for example related to signalling. Indeed, a high noise level has been associated to partial or complete loss of cellular functions [1–3], and there is evidence of evolutionary selection against cellular noise [4, 5]. On the other hand, molecular noise can be beneficial in various circumstances [6]. It can be exploited to drive genetically identical cells to different cell fates in multi-cellular organisms [7–13], or it can induce the phenotypic diversification at the basis of bet-hedging strategies that protect microbial cell populations from sudden environmental changes [14–20].

Focusing specifically on the gene expression process, two possible sources of fluctuations can be defined, i.e. intrinsic and extrinsic noise. Intrinsic noise arises from the inherently stochastic nature of the molecular reactions involved in the transcription, translation, and degradation of messenger RNAs (mRNAs) and proteins. Extrinsic noise is instead the result of fluctuations in global cellular factors such as the concentration of key macromolecules (e.g. ribosomes and polymerases) involved in the process performing catalytic/enzymatic activity [21, 22]. These extrinsic fluctuations can also arise from cell-to-cell differences in metabolic states [23], cellular signalling [24, 25], cell-cycle stage [26–28], and other relevant phenotypic traits associated with cell physiology (i.e. cell growth rate, doubling time, volume, etc) [29–35].

Noise propagation through gene regulation is another key source of gene expression noise. In fact, the number of regulatory inputs of a gene is correlated with its expression variability in *E. coli* [36] and eukaryotes [37, 38], and the topology of the regulatory network can play a crucial role in the extent of noise propagation [39, 40].

A large amount of theoretical and experimental work has focused on disentangling the contributions to protein fluctuations from intrinsic and extrinsic sources (see for example [41–43]). On the other hand, what are precisely the dominant sources of extrinsic noise, and thus how they have to be correctly inserted in effective models of gene expression are still open questions, even if extrinsic noise actually seems the main noise source for sufficiently highly expressed genes [38, 44, 45].

The specific definition of what is considered extrinsic noise depends on the system one wants to explicitly model (a single gene, a small genetic circuits, the whole cell). We focus on a single gene and, from a modelling standpoint, extrinsic noise can be defined as fluctuations of the parameters of the expression process such as degradation and production rates. All the possible biological sources of extrinsic noise listed above can affect in complex ways one or more parameters. For example, growth rate fluctuations directly impact the dilution rate of proteins through volume fluctuations, but at the same time can change the protein production rates by affecting the concentration of key enzymes, such as ribosomes, that are closely coupled with cell growth [46]. The problem is to pinpoint which are the parameters that are most affected in the biological system of interest, in order to design the correct minimal model of the stochastic process of gene expression.

This work focuses on this problem by looking at the consequences of extrinsic noise on the out-of-equilibrium dynamics of gene expression. This regime represents the dynamical approach to a steady state, during which the reactions of molecule production and degradation are not yet balanced. Specifically, we will provide analytical expressions supported by simulations that characterize how the protein level and its fluctuations evolve during gene activation and inactivation in the presence of extrinsic fluctuations on different parameters. On top of the theoretical interest of this analysis, the results have immediate practical applications. In fact, the different dynamic profiles of protein noise that we characterized naturally provide an experimentally accessible method to distinguish between different extrinsic-noise scenarios.

## 2. Materials and methods

### 2.1. An effective model of stochastic gene expression with extrinsic noise

**Figure 1.**
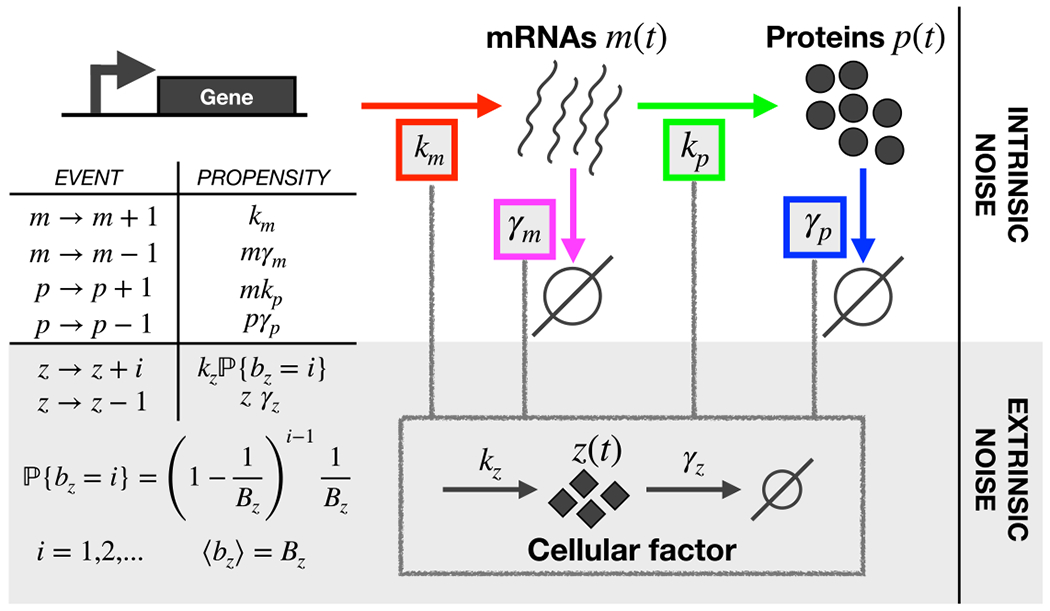
Model of the gene expression process with extrinsic noise. Extrinsic noise is included in a basic two-step model of stochastic gene expression by introducing the cellular factor z(t). z(t) can affect any parameter of the model: transcription and translation rates ($k_{m}$ and $k_{p}$), as well as degradation rates for mRNAs and proteins ($\gamma_{m}$ and $\gamma_{p}$). The table on the left lists the possible reactions for mRNAs m, proteins p and for the cellular factor z with the respective propensity functions that set the event frequencies.

The ‘standard model’ of stochastic gene expression takes into account messenger RNA and protein production and degradation as first-order chemical reactions [47–50]. After activation, the gene is transcribed by RNA polymerases in mRNAs with a fixed rate $k_{m}$; each mRNA (m) is in turn translated into proteins (p) with rate $k_{p}$. Proteins and mRNAs are also removed at specific constant rates ($\gamma_{m}$ and $\gamma_{p}$). Figure 1 schematically represents this set of reactions.

The degradation rate of mRNA molecules sets their average lifetime $\left(1/\gamma_{m}\right)$, which is typically short compared to the average residence time of proteins $\left(1/\gamma_{p}\right)$. Especially in microorganisms, the average lifetime of mRNAs is just few minutes [44]. On the other hand, the rate $\gamma_{p}$ is mainly set by dilution due to cell growth and division [44, 50–52], and thus the protein lifetime is essentially set by the cell doubling time $\tau_{p}=ln(2)/\gamma_{p}$ [53].

The master equation describing this simple two-step model of gene expression (figure 1) can be solved assuming that the promoter is activated at time t=0 and the initial number of mRNAs and proteins is zero [50]. In particular, the dynamics of the average protein numbers is described by

(1) $$\langle p(t)\rangle =p_{ss}\left(\frac{\gamma_{p}\left(1-e^{-\gamma_{m}t}\right)-\gamma_{m}\left(1-e^{-\gamma_{p}t}\right)}{\gamma_{p}-\gamma_{m}}\right)$$

where $p_{ss}=k_{m}k_{p}/\gamma_{m}\gamma_{p}$ is the average protein level at steady state. The noise can be quantified by the coefficient of variation squared ${CV}_{p}^{2}=\sigma_{p}^{2}/{\langle p\rangle }^{2}\equiv \eta^{2},$ where $\sigma_{p}^{2}$ is the variance of the protein number. The coefficient of variation ${CV}_{p}$ represents the relative fluctuations, and it is an intuitive and dimensionless measure that can be directly compared with experimental values.

For stable proteins, the timescale separation between the dynamics of mRNAs and proteins $\left(\gamma_{m}\gg \right.\gamma_{p}$) can be used to derive a compact expression for the time evolution of the intrinsic noise [50, 54] and for its equilibrium value:

(2) $$\eta_{Int}^{2}(t)=\frac{1}{\langle p(t)\rangle }\left(1+B_{p}+B_{p}e^{-\gamma_{p}t}\right)\;\;\;\;\overset{\text{t}\to \infty }{\to }\frac{1}{p_{ss}}\left(1+B_{p}\right)\equiv \eta_{Int,ss}^{2}.$$

$B_{p}=k_{p}/\gamma_{m}$ is the protein burst size, i.e. the average number of proteins produced by a single mRNA during its lifetime. As the expression at steady state shows, burstiness introduces an amplification factor with respect to Poisson noise. We simulate realistic and relatively high levels of expression ($p_{ss}=2000$ proteins in most examples we will describe) with sufficiently low protein burst sizes (few units). The goal is to focus on genes for which the intrinsic contribution to expression noise is not dominant with respect to the extrinsic part, at least at steady state. Large-scale experimental studies in both *E. coli* and yeast have suggested that this is the typical case for highly expressed genes by looking at the scaling of protein noise with the average protein level [38, 44]. Interestingly, a similar scaling seems to hold also for mRNA fluctuations [55].

We will also consider the dynamics of a gene at steady state that is inactivated at the transcriptional level. In this case, the average protein evolution in time is given by

(3) $$\langle \tilde{p}(t)\rangle =p_{ss}\left(\frac{\gamma_{p}\left(e^{-\gamma_{m}t}\right)-\gamma_{m}\left(e^{-\gamma_{p}t}\right)}{\gamma_{p}-\gamma_{m}}\right).$$

So far we have only included intrinsic fluctuations since all the rates were constant. To include extrinsic fluctuations, we introduce a generic cellular factor z that can affect production or degradation rates (figure 1). For example, fluctuations in RNA polymerases or ribosomes will be captured by a direct action of the factor z on the production rates $k_{m}$ or $k_{p}$. More generally, the cellular factor z can capture the consequences that fluctuations in cell physiology can have on gene expression by modulating the affected rates.

Extrinsic fluctuations often have a lifetime that is not negligible and can be comparable to the cell cycle and to protein half-life [21, 44, 56]. Therefore, extrinsic noise is typically referred to (and modelled as) ‘colored’ noise [43], which is a noise with a characteristic timescale. In our context, this timescale depends on the effective (and often unknown) source of extrinsic fluctuations.

To explore the role of both extrinsic fluctuations strength and timescale, we model the dynamics of the cellular factor as a bursty birth-and-death process with constant production and degradation rates ($k_{z}$ and $\gamma_{z}$). With this modelling choice, production events happen at a constant rate $k_{z}$ (as in a Poisson process) and the burst size is $b_{z}$, a random variable sampled by a geometric probability distribution with average burst size $B_{z}$ [42]. Tuning $k_{z}$, $\gamma_{z}$ and $B_{z}$, the extent $\left({CV}_{z}\right)$ and the timescale $(\tau_{z}=\text{ln}(2)/\gamma_{z})$ of extrinsic fluctuations can be independently modulated.

We can thus introduce extrinsic noise on any biochemical rate by multiplying its value to the cellular factor. In other words, we can substitute any parameter θ of the system with $\theta \to \theta (t)=\theta \frac{z(t)}{\langle z\rangle }$. In this way, the average parameter value is still θ, i.e. the value set in the absence of extrinsic fluctuations, but it fluctuates according to z.

The intrinsic noise $\eta_{Int}$ will be defined as the variability associated with the model of stochastic gene expression with constant parameters. Instead, the extrinsic noise $\eta_{Ext}$ can be quantified as the difference between the total measured protein variability and the intrinsic part.

### 2.2. Assessment of the time evolution of protein cell-to-cell variability

The definition of intrinsic and extrinsic noise naturally implies the decomposition $\eta^{2}=\eta_{Int}^{2}+\eta_{Ext}^{2}$ [57]. This decomposition is also valid out-of-equilibrium and for each parameter θ affected by extrinsic fluctuations of strength defined by ${CV}_{z}$ and timescale by $\tau_{z}$. Therefore, we can write

(4) $$\eta_{\theta }^{2}\left(t;{CV}_{z},\tau_{z}\right)=\eta_{Int}^{2}(t)+\eta_{\theta ,Ext}^{2}\left(t;{CV}_{z},\tau_{z}\right).$$

We are interested in the dynamics of gene-expression noise approaching a steady state. In the case of fluctuations of the production rates $k_{m}$ or $k_{p}$, it is possible to derive the exact transient protein noise expression by solving the corresponding system of ordinary differential equations. The details of the calculation can be found in the appendix, but the main result is that for extrinsic noise acting on production rates we can provide an analytical estimate of the time evolution of protein noise.

Unfortunately, the same approach cannot be applied when extrinsic fluctuations affect the dilution rate $\gamma_{p}$. Nevertheless, an approximate expression for the steady-state protein noise $\eta_{\gamma_{p},ss}^{2}\left({CV}_{z},\tau_{z}\right)$ can still be calculated as a function of the timescale and strength of extrinsic noise. In order to provide also an expression for the out-of-equilibrium noise, we can assume that the time dependence in the noise expression can be factorized as

(5) $$\eta_{\theta ,Ext}^{2}\left(t;{CV}_{z},\tau_{z}\right)≃F_{\theta }(t)G_{\theta }\left({CV}_{z},\tau_{z}\right).$$

$F_{\theta }(t)$ explicitly captures the time-dependent part of the impact of θ fluctuations on gene expression noise, while $G_{\theta }\left({CV}_{z},\tau_{z}\right)$ is time independent and takes into account the features of extrinsic noise. Assuming this factorization, an intuitive although approximate expression can be provided using the framework of sensitivity analysis [58]. Basically, we can first evaluate the fluctuations due to the extrinsic factor acting on the parameter θ at steady state, and this is possible for every choice of θ. We can then further assume that the effect of the fluctuating parameter θ on the protein level is predominantly set by the first-order dependency of the average protein dynamics on θ. In other words, we can estimate the protein variance in time using the propagation of uncertainty as

(6) $$\sigma_{p}^{2}(t)≃{\left(\frac{\partial \langle p(t)\rangle }{\partial \theta }\right)}^{2}\sigma_{\theta }^{2}\left({CV}_{z},\tau_{z}\right).$$

$\sigma_{\theta }^{2}\left({CV}_{z},\tau_{z}\right)$ is the variance of the parameter θ, which is given by the variance of the extrinsic factor z. The relation can be rephrased for the coefficient of variation as

(7) $$\eta_{\theta ,Ext}^{2}(t)≃{\left(\frac{\theta }{\langle p(t)\rangle }\frac{\partial \langle p(t)\rangle }{\partial \theta }\right)}^{2}{CV}_{\theta }^{2}\left({CV}_{z},\tau_{z}\right)\;\;\;\;\;≃{\left(\frac{\theta }{\langle p(t)\rangle }\frac{\partial \langle p(t)\rangle }{\partial \theta }\right)}^{2}{CV}_{z}^{2},$$

where, by definition, ${CV}_{\theta }^{2}={CV}_{z}^{2}$ at any given time.

This estimate provides the functional dependency of $F_{\theta }(t)$, while the time-independent noise $CV_{z}^{2}$ can be included in the factor $G_{\theta }\left({CV}_{z},\tau_{z}\right)$ of the decomposition in equation (5). The approximation considers p(t) and θ as continuous variables and neglects the impact of the strength and timescale of extrinsic fluctuations on the time dependent factor.

From the expression of p(t), it is easy to show that ${\text{lim}}_{t\to \infty }F_{\theta }(t)=1$ for any parameter θ. This crucial observation implies that, in order to have consistency at steady state, the factor $G_{\theta }\left({CV}_{z},\tau_{z}\right)$ has to be equal to the extrinsic noise at steady state $\eta_{\theta ,Ext,ss}^{2}$.

Therefore, we finally have explicitly defined the two factors of equation (5) as

(8) $$\;\;\;F_{\theta }(t)={\left(\frac{\theta }{\langle p(t)\rangle }\frac{\partial \langle p(t)\rangle }{\partial \theta }\right)}^{2};\;G_{\theta }\left({CV}_{z},\tau_{z}\right)=\eta_{\theta ,Ext,ss}^{2}\left({CV}_{z},\tau_{z}\right).$$

As discussed above, analytical expressions can be calculated for the total protein noise at steady state $\eta_{\theta ,ss}^{2}$ as a function of the strength and the timescale of extrinsic fluctuations (see equations (A15), (A18) and (A22)). Analogously, the intrinsic noise $\eta_{Int,ss}^{2}$ can be calculated [50]. Therefore, the extrinsic part can be extracted with a simple subtraction $\eta_{\theta ,Ext,ss}^{2}\left({CV}_{z},\tau_{z}\right)=\eta_{\theta ,ss}^{2}-\eta_{\mathrm{Int,ss}}^{2}$

All the analytical expressions have been tested with extensive numerical simulations using the exact Gillespie algorithm [59] as detailed in the appendix (section ‘Stochastic simulation specification’). In the following sections the analytical curves will always be supported by and compared to numerical results. More specifically, for each model configuration, we generated 5 × 10^3^ trajectories simulating the reactions reported in figure 1. In order to estimate the confidence interval of our measurements of noise, we first calculated the protein coefficient of variation values for 5 independent sets of 10^3^ trajectories. The variability of the different values obtained is comparable or smaller than the symbol size used in the figures. We also calculated the interval corresponding to the 95% confidence level for the coefficient of variation values obtained with the bootstrap method [60]. Again, the error interval is consistently smaller than the marker symbols and thus not explicitly shown in the figures.

## 3. Results

### 3.1. Extrinsic fluctuations of the protein dilution rate alter the average protein dynamics

The first result of this work is to provide analytical expressions for the protein dynamics and its variability in the presence of extrinsic fluctuations acting on different gene expression parameters. These analytical results are detailed in the Methods section and in the appendix and will be discussed in the following sections.

Here, we focus on the expected value of the protein level at steady state for different extrinsic noise sources. The different expressions we obtained show that extrinsic fluctuations acting on production rates (i.e. translation rate $k_{p}$ and transcription rate $k_{m}$) do not alter the average protein level $p_{ss}$ described by equation (1) at equilibrium, but fluctuations of the dilution rate $\gamma_{p}$ can significantly change it (equation (A21)). When the extrinsic noise acts on the dilution rate, the system of differential equations describing the protein moment dynamics is not closed, but an approximate moment-closure technique (see appendix, section ‘Protein dilution rate fluctuations’, equation (A20)) can be used to estimate the expected protein level at steady state as

(9) $$p_{\gamma_{p},ss}\left({CV}_{z},\tau_{z}\right)\;\;≃\frac{p_{ss}}{2}\left[\sqrt{{\left(1+\frac{\tau_{z}}{\tau_{p}}\right)}^{2}+4\frac{\tau_{z}}{\tau_{p}}{CV}_{z}^{2}}+\left(1-\frac{\tau_{z}}{\tau_{p}}\right)\right].$$

The expression above indicates how the protein level depends on the cellular factor z and how it is different by the value $p_{ss}=k_{m}k_{p}/\gamma_{m}\gamma_{p}$ without extrinsic noise. In particular, it increases with the fluctuation strength ${CV}_{z}$ and has a sigmoidal dependence on the fluctuation timescale $\tau_{z}$. These analytical predictions are well supported by stochastic simulations (appendix figure A2).

Therefore, the noise-induced alteration of the average behaviour, also known as deviant effect [40, 61], generates a discrepancy with respect to the classic deterministic prediction $p_{ss}$, and this discrepancy grows with the level of extrinsic fluctuations. Thus, deviations from the expected average protein value could in principle be used in experimental settings as hallmarks of large extrinsic fluctuations acting on degradation rates. However, this observation would practically require the knowledge of the process parameter values, which are often not known.

This work aims to characterize the complex interplay between extrinsic fluctuations and protein dynamics in single cells. We will show that this characterization is instrumental to define easily measurable signatures of the possible dominant sources of extrinsic noise in a system, even when the specific parameter values are not known. However, in order to do so, we need to compare the expected single-cell protein dynamics with extrinsic fluctuations acting on different parameters with a fixed common average steady-state value. As explained in detail in the appendix (section ‘A mathematically controlled comparison’) and shown in figure A3, we implemented this classic ‘mathematically controlled comparison’ [52, 62] by taking into account the deviant effects and constraining the average steady-state protein value for any magnitude and timescale of extrinsic fluctuations, independently of which the noisy parameter is.

### 3.2. The dynamics of cell-to-cell variability is strongly dependent on the dominant source of extrinsic noise

**Figure 2.**
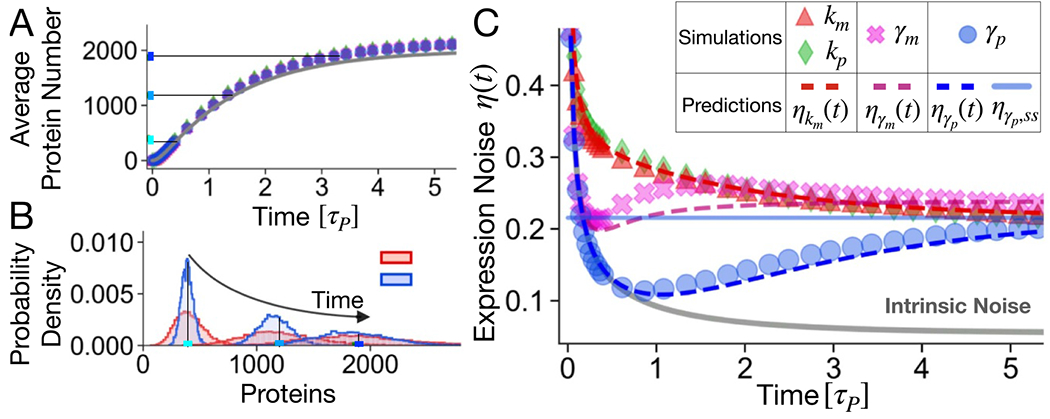
Cell-to-cell variability for different sources of extrinsic noise. We compare the time evolution of expression variability during the activation dynamics when extrinsic fluctuations affect a single rate of production ($k_{m}$, $k_{p}$) or of degradation ($\gamma_{m}$ or $\gamma_{p}$). Even if the extrinsic noise properties are fixed (${CV}_{z}=0.3$, $\tau_{z}/\tau_{p}=1$, ⟨z⟩=1000 copies), different protein noise profiles η(t) can be observed depending on the fluctuating parameter. (A) Thanks to the controlled comparison, the average protein level approaches a fixed steady state following equation (1), independently of the source of extrinsic noise. (B) The protein probability densities are qualitatively different in the presence of fluctuations of $k_{m}$ or $\gamma_{p}$. The mean protein levels at different times are reported as vertical lines and correspond to the horizontal lines in (A). (C) The total gene expression noise, quantified by the coefficient of variation, is reported as a function of time during the transient regime (time $\in [0;6]\tau_{p}$). The continuous grey line represents the variability without extrinsic noise ${CV}_{z}=0$, i.e. only the intrinsic noise described by equation (2). The horizontal blue line marks the approximate prediction for the steady-state expression variability under fluctuations of $\gamma_{p}$, to which the values of the simulations asymptotically tend. The dashed lines correspond to the theoretical predictions (equations (10) and (14)), which are well compatible with the simulation results (symbols).

This section describes the single-cell expression dynamics when a gene is activated in the presence of extrinsic fluctuations. The main observation is that the protein noise dynamics is qualitatively different depending on which parameter is predominantly affected by extrinsic fluctuations. In fact, even if the average protein dynamics is constrained to be the same (figure 2(A)), as explained in the previous section, the protein probability densities are significantly different at short times depending on the extrinsic noise source (figure 2(B)). The out-of-equilibrium protein fluctuations are higher for noise affecting production rates rather than degradation rates. This distinction cannot be done at equilibrium since the probability densities progressively collapse as the protein level approaches the steady state.

Figure 2(C) explicitly reports the dynamics of protein expression noise, which shows different behaviors depending on the dominant source of extrinsic noise. While we used the ${CV}^{2}$ as the noise measure in our mathematical expressions, the coefficient of variation (η(t)=CV) is reported in figure 2(C) and in the following figures as a more intuitive measure. The trends in the presence of fluctuations of the production rates (i.e. $k_{m}$ or $k_{p}$) are qualitatively and quantitatively similar. The simulations are well explained by the exact formula (fully derived in the appendix sections ‘Transcription burst frequency fluctuations’ and ‘Translation rate fluctuations’) for the time-evolution of protein noise in the presence of transcription rate fluctuations (red dashed line in figure 2):

(10) $$\eta_{k_{m}}^{2}\left(t;{CV}_{z},\tau_{z}=\tau_{p}\right)=\frac{1}{{\left(e^{\gamma_{p}t}-1\right)}^{2}}\;\;\times \left[\left(e^{2\gamma_{p}t}-1\right)\eta_{Int}^{2}(t)+\frac{1}{2}\left(e^{2\gamma_{p}t}-2\gamma_{p}t-1\right){CV}_{z}^{2}\right].$$

The corresponding steady state limit is given by

(11) $$\underset{t\to \infty }{\text{lim}}\eta_{k_{m}}^{2}\left(t;{CV}_{z},\tau_{z}=\tau_{p}\right)=\eta_{k_{m},ss}^{2}\left({CV}_{z},\tau_{z}=\tau_{p}\right)\;\;\;\;\;\;\;\;\;\;\;=\eta_{Int,ss}^{2}+\frac{{CV}_{z}^{2}}{2}.$$

The time dependence of equation (10) is analogous to the one of intrinsic fluctuations, given by equation (2) and corresponding to the grey line in figure 2(C). The presence of extrinsic noise essentially increases the relaxation point to the higher noise value predicted by equation (11) without changing the monotonous decreasing trend.

This result can be intuitively understood by considering the factorization proposed in equation (5). Since $\partial \langle p(t)\rangle /\partial k_{m}=\langle p(t)\rangle /k_{m}$ and $\partial \langle p(t)\rangle /\partial k_{p}=\langle p(t)\rangle /k_{p}$, the factors $F_{k_{m}}(t)$ and $F_{k_{p}}(t)$ do not depend on time, thus explaining why $\eta_{k_{m}}(t)$ and $\eta_{k_{p}}(t)$ qualitatively follow the intrinsic noise profile. Indeed, the extrinsic fluctuations uniformly affect gene expression during the activation dynamics by shifting the total noise in protein level to higher values.

Note that the predicted steady-state levels of fluctuations are all similar in this setting. In principle, fluctuations on different production rates can produce slightly different levels of steady-state protein noise. In fact, fluctuations of the translation rate (and thus of the protein burst size) lead to higher protein noise, as the comparison between their analytical expressions in equations (11) and (A18) shows. However, the additional noise term has a factor $1/p_{ss}$ that makes it negligible for highly expressed genes. Analogously, as explained in details in the appendix (section ‘Protein dilution rate fluctuations’), the estimated value of $\eta_{\gamma_{p},ss}^{2}$ for fluctuations of the degradation rate has a particularly compact form when the timescale of extrinsic fluctuations approximately matches the intrinsic one (as in the example considered):

(12) $$\eta_{\gamma_{p},ss}^{2}\left({CV}_{z},\tau_{z}=\tau_{p}\right)≃\eta_{Int,ss}^{2}+\left[\sqrt{1+{CV}_{z}^{2}}-1\right].$$

The Taylor expansion of the extrinsic contribution for small ${CV}_{z}^{2}$ is ${CV}_{z}^{2}/2$, precisely as in the case of fluctuations on production rates (equation (11)).

On the other hand, the protein noise dynamics η(t) displays a qualitatively different and nonmonotonic trend for fluctuations of degradation rates ($\gamma_{m}$ or $\gamma_{p}$). In particular, extrinsic fluctuations on $\gamma_{p}$ do not affect the noise dynamics at short times. The total noise is dominated by the intrinsic part as the simulations (blue circles) precisely lay on the intrinsic noise theoretical prediction (grey line) for $t<\tau_{p}$ in figure 2(C). As long as the number of proteins is small, the degradation term is negligible and proteins accumulate approximately linearly with a slope that does not depend on $\gamma_{p}$. This can be proven by considering the Taylor expansion for t→0 of ⟨p(t)⟩ when $\gamma_{m}\gg \gamma_{p}:$

(13) $$\langle p(t)\rangle \approx p_{ss}\left(1-e^{-\gamma_{p}t}\right)\underset{t\to 0}{\approx }p_{ss}\left(1-\left(1-\gamma_{p}t+O\left(t^{2}\right)\right)\right)\;\;\;\;=\frac{k_{m}k_{p}}{\gamma_{m}}t+O\left(t^{2}\right),$$

where we explicitly report the linear term of the expansion, and collect the remaining smaller terms using the standard O notation.

The contribution of $\gamma_{p}$ on the protein dynamics, and thus also on the fluctuations, becomes significant only at sufficiently long times. In a similar way, the impact of the cellular factor on $\gamma_{m}$ is negligible at the beginning of the simulation and grows in time with 〈m(t)〉.

The generalization of this simple argument allows us to roughly quantify the impact of extrinsic fluctuations at any time through the factorization explained in the Methods section. When extrinsic fluctuations affect the protein dilution rate $\gamma_{p}$, our approximate prediction for the time-evolution of protein noise is

(14a) $$\eta_{\gamma_{p}}(t)≃\sqrt{\eta_{Int}^{2}(t)+\eta_{\gamma_{p},Ext}^{2}(t)}$$

(14b) $$\eta_{\gamma_{p},Ext}^{2}(t)≃{\left(\frac{\gamma_{p}}{\langle p(t)\rangle }\frac{\partial \langle p(t)\rangle }{\partial \gamma_{p}}\right)}^{2}\eta_{\gamma_{p},Ext,ss}^{2}.$$

This approximation is displayed as a dashed blue line in figure 2(C) and well captures the results of exact simulations.

In the case of fluctuations of $\gamma_{m}$ we tested the validity of the approach summarized in equation (8) without explicitly calculating the noise at steady state. Thus, the experimental value at steady state was used to obtained the dynamics represented in figure 2(C) as a dashed magenta line. Also in this case the approximation captures the empirical trend.

**Figure A1.**
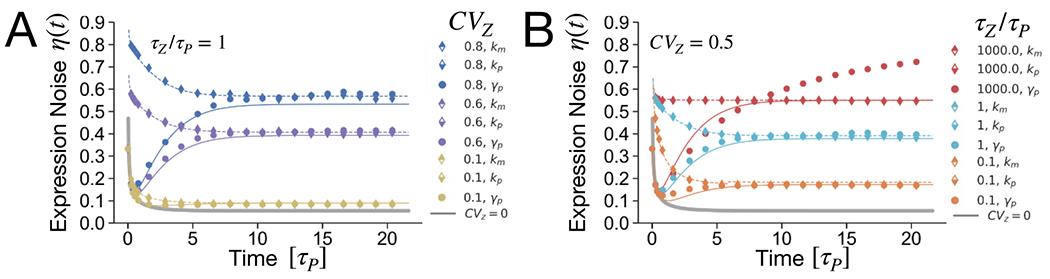
Time evolution of cell-to-cell variability depends on the source of extrinsic noise. The total gene expression noise is reported as a function of time during the activation dynamics; each color represents a specific value of ${CV}_{z}$ (A) or $\tau_{z}/\tau_{p}$ (B), while different line-styles correspond to different fluctuating parameters. The continuous grey line represents the total variability that we observe in the system if ${CV}_{z}=0$, i.e. the intrinsic noise. The dashed lines correspond to the theoretical predictions for fluctuations of the transcription rate (equation (A14)), they are in good agreement with the results of the simulations. The approximate predictions for expression variability under fluctuations of the protein dilution rate is marked by the continuous lines and correspond to equation (A24).

**Figure A2.**
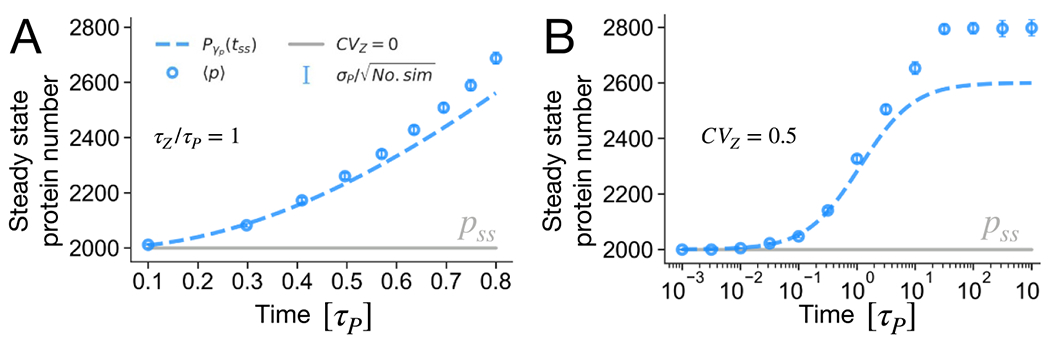
The strength and the timescale of extrinsic fluctuations of the protein dilution rate affect the steady-state level of expression. Functional dependence of protein steady-state level on the extrinsic noise strength (A) and timescale (B), the dashed lines represent the approximate analytical predictions for $p_{\gamma_{p}}\left(t_{ss}\right)$ in case of extrinsic fluctuations acting on the protein dilution rate (equation (A21)). The light blue symbols report the outcomes of our simulations, with circles representing average values and bars standard errors (i.e. the ratio between the standard deviation and the square root of the number of simulations).

**Figure A3.**
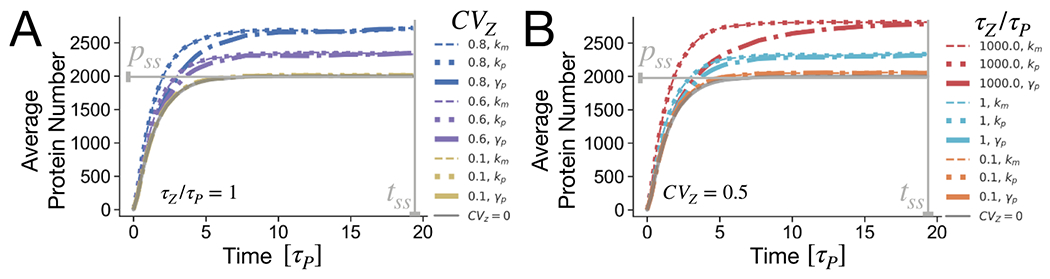
Protein expression tends asymptotically to the same equilibrium level. At time t=0 transcription begins and the gene is turned on. The average protein level of the simulations approaches the steady-state $p\left(t_{ss}\right)$, which depends on the characteristics of the extrinsic source of noise. Due to the appropriate tuning of the parameters, when the cellular factor affects $k_{m}$ or $k_{p}$ the protein expression tends to the same equilibrium level observed for fluctuations of $\gamma_{p}$. (A) The average protein activation dynamics for $\tau_{p}/\tau_{z}=1$ is reported as a function of time (in units of the response-time), each color represents a specific value of ${CV}_{z}$ and different line-styles correspond to different sources of extrinsic noise. (B) The average protein activation dynamics for ${CV}_{z}=0.5$ is reported as a function of time, each color represents a specific value of $\tau_{z}$ (in units of $\tau_{p}$).

The minimum values of $\eta_{\gamma_{m}}(t)$ and $\eta_{\gamma_{p}}(t)$ mark the times at which extrinsic fluctuations start to significantly affect the expression noise. As intuitively expected, this happens approximately at the corresponding molecule lifetimes since they set the timescales of the approach of the steady state. In particular, the transition is at a time ≃1 (in units of $\tau_{p}$) in figure 2(C) for noise on $\gamma_{p}$, and earlier for noise on $\gamma_{m}$ since the mRNAs approaches the steady state quickly. Figure 2 refers to a particular choice of ${CV}_{z}$ and $\tau_{z}=\tau_{p}$ (matched timescales), but the trends are robust with respect to the values of ${CV}_{z}$ and $\tau_{z}$, as reported for example in figure A1.

A relevant implication of this analysis is that the simple observation of the dynamics of protein noise, which is experimentally accessible, can clearly distinguish between alternative sources of gene-expression variability, even if the parameter values are not known.

### 3.3. Linear increase of protein noise with the strength of extrinsic fluctuations

**Figure 3.**
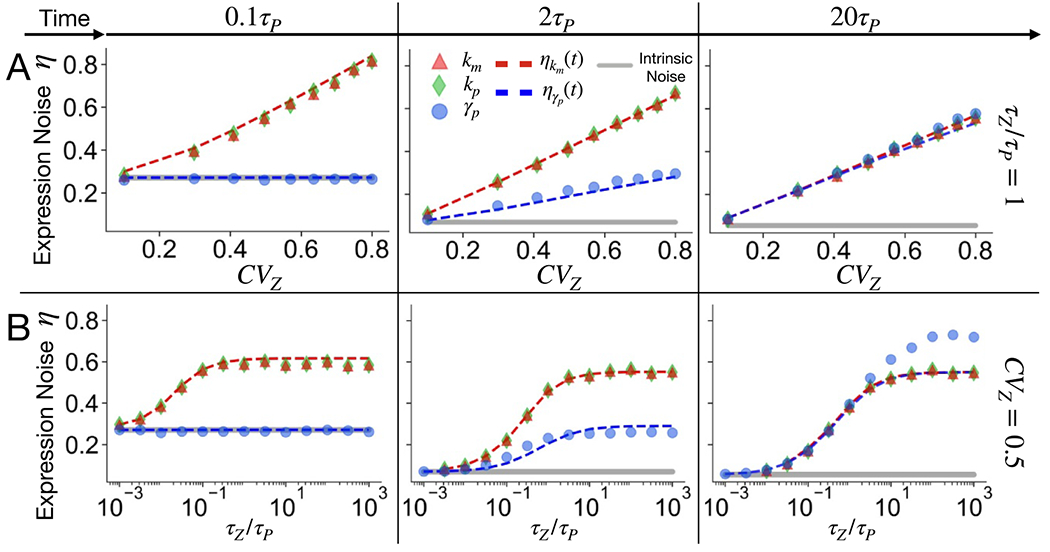
Functional dependence of protein variability on the extrinsic noise strength and timescale. The protein noise is reported as a function of the strength (A) and timescale (B) of extrinsic fluctuations. Different symbols correspond to fluctuations of different expression parameter ($k_{m}$, $k_{p}$ or $\gamma_{p}$) as explained in the legend. The analysis is reported at three different times in the activation dynamics, representing the early stage, an intermediate stage and essentially the steady state. Dashed lines represent the theoretical predictions of equation (10) (red) and equation (14) (blue) that well capture the results of Gillespie simulations. To investigate the role of ${CV}_{z}$ (A), the timescale of z(t) is fixed. In this example it matches the timescale of p(t), i.e. $\tau_{z}=\tau_{p}$. On the other hand, to explore the role of the timescale $\tau_{z}$ (B), the coefficient of variation of the cellular factor has been held constant to ${CV}_{z}=0.5$.

This section explores in detail the role of the strength of extrinsic fluctuations in shaping the single-cell expression dynamics. To model the biologically relevant range of extrinsic fluctuations $\left({CV}_{z}\in [0.1;0.8]\right)$, we considered the data reported in [44] about the noise level of highly expressed protein in *E. coli*. Figure 3(A) reports the protein noise η(t) as a function of the extrinsic noise ${CV}_{z}$. During the early stages of the protein activation dynamics (time $t=0.1\tau_{p}$) the intrinsic component of expression noise (grey line) is significant, and in particular it is the only contribution to the total noise when extrinsic fluctuations act on $\gamma_{p}$. For sufficiently long times, the intrinsic noise becomes less relevant, and the expression variability starts to increase linearly with the strength of extrinsic fluctuations. The proportionality coefficient depends on the fluctuating parameter and on the time of the measurement. The figure refers to the biologically relevant situation of approximately matched timescale $\tau_{z}=\tau_{p}$. In this case, the cellular factor z can represent a protein whose fluctuations affect the production or the degradation of a protein of interest p with a similar average lifetimes (for example set by the cell doubling time). In these conditions, η reaches a single steady-state level for long times ($t=20\tau_{p}$) for any choice of the fluctuating parameter, as displayed by the overlap of the different curves in the right panel of figure 3(A).

The dashed lines in figure 3(A) correspond to our theoretical predictions for $\eta_{k_{m}}$ and $\eta_{\gamma_{p}}$, i.e. respectively equations (10) and (14), and the plots show the agreement with simulation results. Again, we are comparing systems that converge to similar steady state levels of noise. The difference grows in principle with ${CV}_{z}$, but it is negligible within our range of parameter exploration. This can be shown by comparing the Taylor expansion of equation (12) for small ${CV}_{z}^{2}$ to equation (11).

### 3.4. Sigmoidal dependence of protein noise on the timescale of extrinsic fluctuations

The specific timescale of extrinsic fluctuations crucially depends on their biological origin and can be slow or fast with respect to the relevant timescale of protein halflife. For instance, slow fluctuations of a transcription factor level would imply extrinsic fluctuations acting on the transcription rate with a relatively long timescale $\left(\tau_{z}/\tau_{p}\gg 1\right)$, thus making the promoter slowly switch between different states of activity.

Figure 3(B) displays the role of the timescale of extrinsic fluctuations in determining the time evolution of protein variability. The consequences of adding stochasticity through the coupling with an extrinsic factor z(t) are minimal when its autocorrelation time is much smaller than the duration of a typical intrinsic fluctuation of p(t), which is set by the protein lifetime. In fact, when these two timescales are well separated, i.e. $\tau_{z}\ll \tau_{p}$ or equivalently $\gamma_{z}\gg \gamma_{p}$, extrinsic fluctuations are averaged out in the protein dynamics and the extrinsic component is negligible. Indeed, all noise curves start from the intrinsic noise baseline (grey line) for relatively fast z fluctuations in figure 3(B).

In the opposite setting (i.e. $\tau_{z}\gg \tau_{p}$ or $\gamma_{z}\ll \gamma_{p}$), the extrinsic factor evolves slowly in time. Therefore, every cell in the population has a specific random value for the fluctuating parameter that is essentially frozen during the protein dynamics. The system is essentially subjected to what is typically called quenched disorder in statistical physics. Around $\tau_{z}/\tau_{p}>10$ the system enters into this ‘quenched regime’ and the noise η saturates at a value that only depends on the level ${CV}_{z}$ of the quenched noise. The presence of these two regimes makes the dependence on the extrinsic-noise timescale sigmoidal and the crossover between the two regimes is at $\tau_{z}≃\tau_{p}$ as intuitively expected.

As mentioned before, at the beginning of the dynamics $\left(t=0.1\tau_{p}\right)$, intrinsic noise is dominant when the fluctuating parameter is $\gamma_{p}$ (blue circles), and thus the extrinsic contribution to η is negligible independently of its timescale. This is not the case for fluctuations in production parameters. The discrepancy is well capture by our analytical predictions (dashed lines). For longer times, such as $t=2\tau_{p}$, the cell-to-cell variability continuously moves from a dominant intrinsic noise for short living extrinsic fluctuations $\left(\tau_{z}\ll \tau_{p}\right)$ to the opposite situation in the quenched regime $\left(\tau_{z}\gg \tau_{p}\right)$, since in this example ${CV}_{z}\gg \eta_{Int,ss}$.

At the steady state ($t=20\tau_{p}$ in the figure) and in the quenched regime, the gene expression variability is essentially set by the distribution of the extrinsic factor z. Our analytical predictions converge for any fluctuating parameter to a single steady-state value given by the expression

(15) $$\eta_{ss}=\sqrt{\eta_{Int,ss}^{2}+{CV}_{z}^{2}}.$$

However, while the analytical expression seem generally accurate, the noise exceeds the prediction in simulations with $\gamma_{p}$ as a fluctuating parameter. This gap shows the limitations of the analytical approximations made in this case and explained in details in the appendix (section Protein dilution rate fluctuations).

The sigmoidal dependence of protein fluctuations on the extrinsic noise timescale suggests the presence of an effective noise filter. To survive the filtering, the effective frequency of extrinsic fluctuations must be of the same order or lower than the frequency of intrinsic fluctuations. In fact, the possible behavior of simple genetic circuits as low-pass filters was previously investigated using frequency domain analysis [63,64]. Specifically, these studies focused on how extrinsic noise on the production rates can cause alterations in the autocorrelation function of the target protein in a frequency-dependent manner. Our results are compatible and generalize these previous findings. A comprehensive comparison is reported in the appendix (section ‘Protein autocorrelation function in the presence of extrinsic noise’).

### 3.5. Fluctuations of protein dilution rate after a transcriptional block enhance expression noise

**Figure 4.**
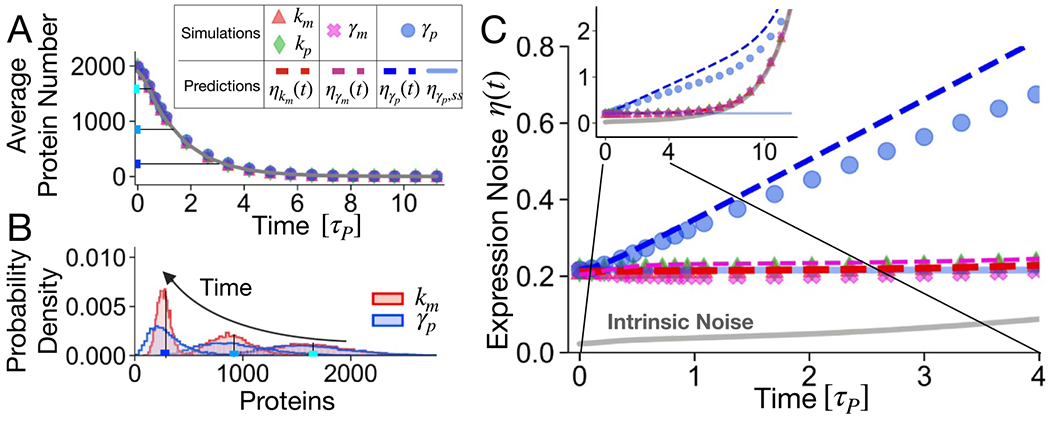
Time-dependent cell-to-cell variability after a sudden transcriptional block. In analogy with figure 2, throughout the four alternative settings, we maintain the inactivation dynamics and we fix the properties of the extrinsic noise. (A) Inactivation dynamics is almost not influenced by extrinsic noise independently of its source, making the controlled comparison method redundant for this particular choice of ${CV}_{z}=0.3$, $\tau_{z}/\tau_{p}=1$, ⟨z⟩=1000 cellular factors. (B) The main differences between proteins probability densities in case of fluctuations of $k_{m}$ or $\gamma_{p}$ are appreciable during the intermediate and the final stage of the transient. (C) The time-evolution of expression variability in case of fluctuations of production rates or mRNA’s degradation rate is similar to the one that we observe in the absence of the source of extrinsic noise (grey continuous line). When fluctuations act on $\gamma_{p}$ their effect is to enhance expression variability, although the general monotonicity is maintained; the subpanel extends the time range to $10\tau_{p}$, showing the exponential growth of the intrinsic noise as the average protein number approaches zero.

The analysis has focused so far on the gene activation dynamics. This section studies the time-evolution of protein noise in the case of a sudden transcriptional repression. We consider a gene expressed at steady-state level whose transcription rate goes to zero at time zero, and we analyze the following protein dynamics when extrinsic noise affects different parameters. Again a mathematically controlled comparison (defined in the Methods section) will be used: the system always start from the same average steady state level as in figure 4(A). Fluctuations acting on different parameters do not alter the average protein decay dynamics (figure 4 (A)), which is well described by equation (3). Analogously, the initial level of fluctuations is given by equations (12) and (11) and is approximately independent on which is the fluctuating parameter in this setting, as it is also shown by the overlap of the protein distributions at the initial time in figure 4(B). Nevertheless, clear differences in the protein probability densities appear as the switch-off dynamics unfolds (figure 4(B)). In particular, extrinsic fluctuations predominantly on the protein degradation rate leads to high-variance protein distributions.

As the expression level progressively approaches zero, the intrinsic contribution grows exponentially, following the grey continuous line in the inset of figure 4(C). After the transcriptional block, the number of mRNAs decays with a timescale set by $\tau_{m}$, which is typically short with respect to the protein lifetime. Therefore, 〈m(t)〉 quickly goes to zero and protein degradation is the only possible reaction left. This explains why the expression noise profiles are equivalent to the intrinsic noise prediction with the exception of $\eta_{\gamma_{p}}(t)$, i.e. when extrinsic fluctuations predominantly affects the protein lifetime.

Our theoretical predictions for η(t) (dashed lines of figure 4(C) derive from the simple argument of equation (8), which when applied in this context gives:

(16) $${\tilde{\eta }}_{\theta }(t)≃\sqrt{{\tilde{\eta }}_{Int}^{2}(t)+{\tilde{\eta }}_{\theta ,Ext}^{2}(t)};$$

(17) $${\tilde{\eta }}_{\theta ,Ext}^{2}\left(t;{CV}_{z},\tau_{z}\right)≃{\tilde{F}}_{\theta }(t)\tilde{G_{\theta }}\left({CV}_{z},\tau_{z}\right);$$

(18) $${\tilde{F}}_{\theta }\left(t\right)={\left(\frac{\theta }{\langle \tilde{p}\left(t\right)\rangle }\frac{\partial \langle \tilde{p}\left(t\right)\rangle }{\partial \theta }\right)}^{2};$$

(19) $$\tilde{G_{\theta }}\left({CV}_{z},\tau_{z}\right)={\tilde{\eta }}_{\theta ,Ext}^{2}(t=0)=\eta_{\theta ,Ext,ss}^{2}.$$

To determine the role of $\tilde{G_{\theta }}\left({CV}_{z},\tau_{z}\right)$, the limit $\lim_{t\to 0}{\tilde{F}}_{\theta }(t)$ has to be considered, which correspond to the initial steady state. In the case of fluctuations of $k_{m}$, $k_{p}$ or $\gamma_{m}$, the term $\tilde{F}(t)$ does not depend on time. Therefore, the noise dynamics is uniformly affected by extrinsic noise. η(t) remains approximately constant until the intrinsic contribution becomes dominant (around $t≃7\tau_{p}$) and makes the noise grow exponentially.

The trend of ${\tilde{\eta }}_{\gamma_{p}}(t)$, i.e. for a fluctuating protein degradation rate, is qualitatively different and well predicted by our analytical estimations. As long as the intrinsic contribution is negligible, protein noise grows linearly with time as ${\tilde{\eta }}_{\gamma_{p}}(t)\approx \left(1+\gamma_{p}t\right)\tilde{\eta }(t=0)$. For sufficient long times, ⟨p(t)⟩ approaches zero and again the noise diverges because of the intrinsic contribution.

The fluctuation trends in the gene deactivation dynamics do not have qualitatively different behaviors as in the case of gene activation, there is a general increase in protein noise [65]. However, an extrinsic noise that mainly affects the degradation rate significantly increases the protein fluctuations immediately after the transcriptional block. On the other hand, it is necessary to wait way longer then the protein lifetime to see a considerable fold in protein noise if the production rates are fluctuating. An estimate of the protein lifetime can be extracted from the average protein dynamics after the transcriptional block. In fact, the average protein value follows equation (3), which is basically an exponential decay with exponent given by the protein lifetime if the protein is stable with respect to the mRNA. These observations can be used in time-coarse experiments to identify the main form of extrinsic noise in the system in analysis.

**Figure A5.**
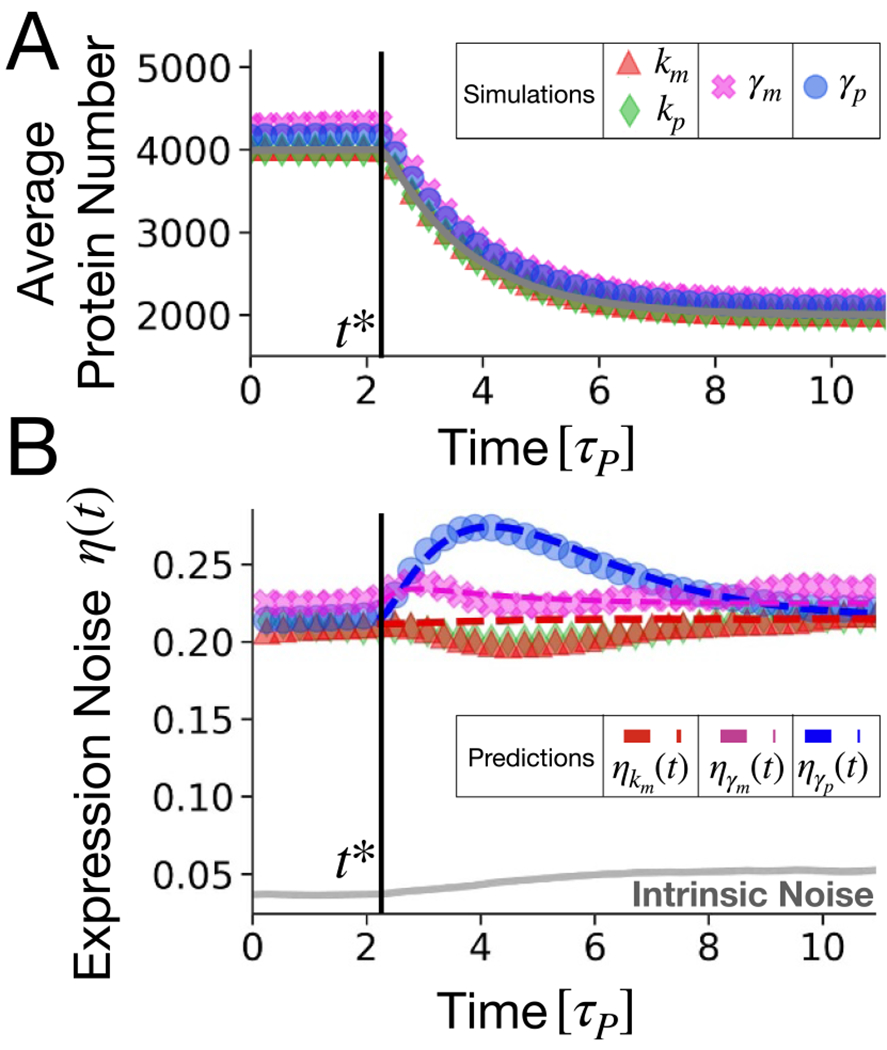
The effect of extrinsic fluctuations during a transition between two steady states. (A) The common dynamics of the average protein number is depicted for different choices of the fluctuating parameter (different symbols). The trend is compatible with equation (A28). (B) The protein variability in time is qualitatively different if extrinsic fluctuations act on the protein degradation rate. This distinctive pattern is accurately predicted by our approximated equations, depicted as dashed lines. Fluctuations in mRNA degradation rate also lead to a similar trend, albeit to a lesser extent (pink dashed line).

As the inset of figure 4(C) shows, for long times the number of proteins becomes small and the intrinsic fluctuations become dominant. To better isolate the effect of extrinsic fluctuations on the noise dynamics, we also considered a sudden shift in protein production. In particular, we analyze the the protein dynamics from a high steady-state level to a lower one, but still far from zero. In this way, the intrinsic fluctuations remain a small contribution across the whole transition. This setting can also be realized experimentally by controlling an inducible promoter to a lower transcription rate. The results of this analysis are reported in the appendix (section ‘The effect of extrinsic fluctuations after a reduction of the transcription rate’), and in particular in figure A5. Also in this case the protein noise dynamics is qualitatively different if the extrinsic noise acts on production rates or on degradation rates.

### 3.6. The interplay between multiple fluctuating parameters

One single parameter could be predominantly affected by extrinsic fluctuations as we have assumed so far. For example, if fluctuations in ribosome concentration are the main source of extrinsic noise, as it has been hypothesized in fast-growing bacteria [44], the translation rate would be the main fluctuating parameter in a corresponding model of stochastic gene expression. However, more general variability in cell physiology can affect multiple expression parameters in different ways. For example, growth rate is coupled to ribosome concentration as well as to cell volume, and thus its fluctuations can influence translation rate as well as protein dilution. Therefore, this section explores the consequences of extrinsic fluctuations affecting two different expression parameters with comparable intensity. In particular, we focus on the illustrative example of fluctuations in the transcription rate and in the protein dilution/degradation rate, and we describe their possible interactions in defining the final level of protein noise.

Previous studies have shown that extrinsic fluctuations can combine constructively or destructively at the steady state depending on their action on parameters [42, 43]. Here, we extend the analysis to the out-of-equilibrium dynamics.

More specifically, a single extrinsic factor z can simultaneously set the fluctuations of the two different parameters in a positively or negatively correlated fashion. Alternatively, two independent sources of noise ($z_{1}$ and $z_{2}$) can individually affect the two different parameters making their fluctuations uncorrelated. While these represent the more clear-cut scenarios, a specific biological system could present multiple sources of extrinsic noise and thus intermediate and more nuanced situations.

**Figure 5.**
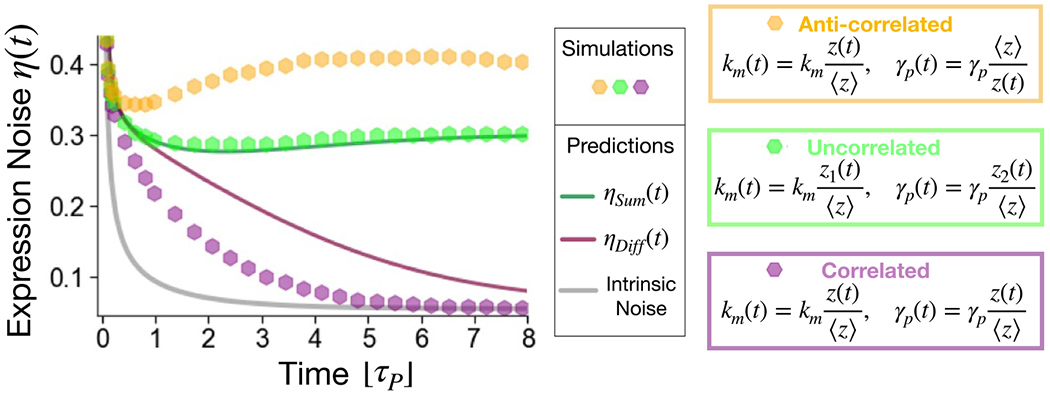
Simultaneous fluctuations of $k_{m}$ and $\gamma_{p}$ can combine constructively or destructively during transient dynamics. We consider the combined action of extrinsic alterations on the transcription rate and the dilution rate. The lines represent our theoretical prediction, while simulation results are reported as hexagonal dots. The grey line corresponds to the analytical intrinsic noise. The fluctuations are either uncorrelated and generated by individual sources of stochasticity, $z_{1}(t)$ and $z_{2}(t)$, (in green), or generated by the same source, z(t), affecting the parameters in a correlated (purple) or anti-correlated way (yellow). The simulation specifications are: $\left\langle z_{1}\right\rangle =\left\langle z_{2}\right\rangle =\langle z\rangle =1000$ cellular factors, ${CV}_{z_{1}}={CV}_{z_{2}}={CV}_{z}=0.3$ and $\tau_{z_{1}}=\tau_{z_{2}}=\tau_{z}=\tau_{p}$.

When two sources of noise independently affect the transcription and degradation rates, a simple additive combination can be observed. In other words, the total extrinsic noise is well described by the sum of the extrinsic contributions we previously calculated with a single fluctuating parameter. Therefore, the total protein noise can be simple written as

(20) $$\eta_{\text{Sum}}(t)=\sqrt{\eta_{Int}^{2}(t)+\left|\eta_{k_{m},Ext}^{2}(t)+\eta_{\gamma_{p},Ext}^{2}(t)\right|},$$

where $\eta_{k_{m},Ext}^{2}(t)$ and $\eta_{\gamma_{p},Ext}^{2}(t)$ are the expressions calculated for $k_{m}$ or $\gamma_{p}$ as the only fluctuating parameter. Figure 5 shows that this analytical prediction fits rather well the simulation results (green dots).

When a single extrinsic factor z sets the two parameters in a correlated way, a positive fluctuation in z increases the protein production rate but, at the same time, also boosts the degradation rate. Therefore, the two fluctuations combine destructively, and the resulting extrinsic contribution is approximately the difference between the two noise contributions. The total protein noise can thus be described by

(21) $$\eta_{\text{Diff}}(t)=\sqrt{\eta_{Int}^{2}(t)+\left|\eta_{k_{m},Ext}^{2}(t)-\eta_{\gamma_{p},Ext}^{2}(t)\right|}.$$

This expression is plotted as a continuous purple line in figure 5 and captures the behavior of the corresponding simulations (purple dots). However, there is a clear quantitative mismatch between simulation and analytical results, indicating that using the difference between the two extrinsic noise sources is an oversimplification and their interplay can be more complex.

On the contrary, when a single source of stochasticity affects $k_{m}$ and $\gamma_{p}$ in an anti-correlated way, their corresponding noise contributions combine constructively, so that the total noise exceeds $\eta_{\text{Sum}}(t)$.

The non-monotonous trend of protein noise, which is the hallmark of dominant extrinsic fluctuations in $\gamma_{p}$ as shown in figure 2, is thus conserved also if another parameter fluctuates with an equivalent variance, as long as the two contributions do not combine destructively.

## 4. Discussion

The analysis of noise has always played a crucial role in our quantitative understanding of basic biological processes [66–69]. More specifically, from the first single-cell experiments with clonal bacterial populations, the substantial cell-to-cell variability in gene expression was evident, and could be traced back to two distinct origins, i.e. intrinsic and extrinsic noise [39, 41]. Experimentally, the dual-color experiment allows to disentangle the two noise factors [41], although their interplay can actually be complex and not trivial to interpret [22]. Large-scale studies showed the major relevance of the extrinsic contribution, especially for sufficiently expressed genes [38, 44, 45]. In parallel, theoretical analysis have been proposed on the consequences of extrinsic noise on protein distributions at steady state [42, 43]. However, understanding the dominant biological sources of extrinsic noise in a specific organism, and thus the expression rates mostly affected by these general cellular factors, has proven to be a difficult task. A major problem is that factors related to cell physiology such as metabolism or cell cycle, which are known to have substantial cell-to-cell variability [23, 70, 71], can affect multiple steps in gene expression from the gene copy number to the translation rate. This makes hard to hypothesize what rates are actually predominantly fluctuating and thus to build appropriate mathematical models.

The observation that equilibrium distributions could not be sufficient to characterize the noise sources was previously recognized in an analysis on the role of RNA degradation fluctuations [72]. Analogously, the idea that the molecule dynamics can provide additional information on the dominant source of noise has been exploited to distinguish between alternative intrinsic noise models [65, 73]. This paper extends these intuitions and provides a detailed theoretical analysis of the possible scenarios in which different parameters are predominantly coupled with an extrinsic noise source. More specifically, we characterized the dynamics of the average protein level and of its fluctuations out-of-equilibrium, showing that the dynamics specifically presents signatures of the extrinsic noise source. Current experimental techniques based on fluorescence time-lapse microscopy [74, 75], potentially coupled with microfluidic devices to keep cells in a controlled environment for many generations [75, 76], give access to the expression dynamics at the single-cell level when a reporter gene is induced or suppressed. Therefore, our results can be directly compared with experimental trends, providing a simple tool to better understand the dominant sources of extrinsic noise by pinpointing the fluctuating expression parameters. We also explored in detail the parameter space, providing analytical estimates of how the picture can quantitatively change as a function of the timescale and strength of extrinsic fluctuations.

We used a simple and general modelling framework to keep it amenable of analytical calculations and applicable to different biological systems. The actual cellular mechanisms giving rise to extrinsic noise are only phenomenologically captured by a generic ‘cellular factor’ z described by a three-parameter distribution of which we varied the parameter values. An alternative (and complementary) approach would be to start from specific mechanistic descriptions of global cellular factors, for biological systems in which are available, and explore their impact on noise in gene expression. For example, in fast growing bacteria quantitative descriptions of the cell cycle [77, 78] and of the ‘laws’ governing the global resource partitioning in the cell [46, 79, 80] have been proposed. These basic aspects of cell physiology greatly contribute to gene expression rates and their fluctuations by setting the cell volume, protein dilution, as well as the concentration of key enzymes [81]. Along this line, recent work focused on the effect of cell cycle and growth rate variability on gene expression noise [33–35]. It would be interesting to analyze the relation between specific mechanistic models of the extrinsic noise source and the statistics of the general factor z in our approach, in order to apply our analytical results and predictions for the out-of-equilibrium dynamics to more detailed biological descriptions.

While this work focuses on the dynamics of an isolated gene, a natural extension will be to consider more complex regulatory interactions, such as the ubiquitous circuits of auto-regulation, feed-forward loops and regulatory cascades. Noise propagation through transcriptional regulation is a known substantial source of extrinsic noise [36, 37], and thus the dynamical fluctuation properties here described could in principle change in the presence of regulatory circuits.

In the framework of synthetic biology, genetic circuits can be designed using different types of regulators, thus introducing controlled modulations of specific rates, from transcription rates to degradation rates [82–84]. The choice of which rate is regulated will naturally induce fluctuations of the corresponding parameter because of the coupling with the regulator fluctuations. Thus, different dominant sources of extrinsic noise can be introduced in synthetic circuits, in principle with a different strength and timescale. Therefore, genetically engineered systems can represent the ideal testing ground for our predictions.

Finally, the presence of extrinsic noise can have relevant consequences on the timing precision of genetic circuits. Models of stochastic timing in gene expression have typically focused on the consequences of intrinsic noise [85–87]. However, characterizing the out-of-equilibrium dynamics with extrinsic noise, the results presented here can be used to derive estimates of first-passage-time distributions in the presence of different types of extrinsic fluctuations.

## Figures and Tables

**Figure A4.**
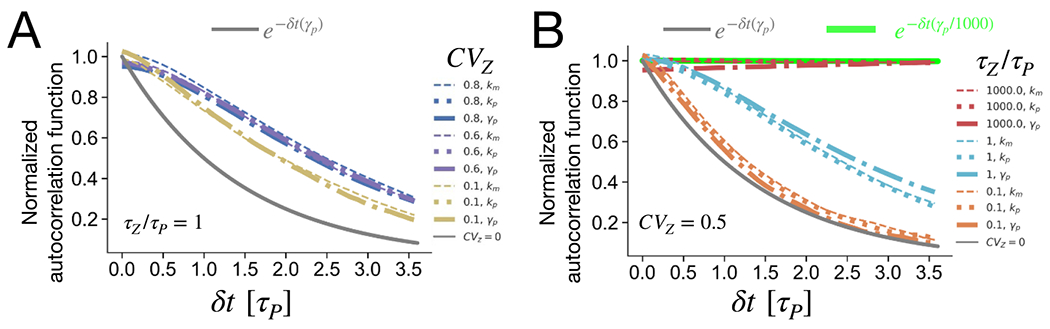
Extrinsic fluctuations affect the autocorrelation time of proteins. Each color represents a particular choice of ${CV}_{z}$ (A) or $\tau_{z}$ (B). The plots show the shift of the autocorrelation function of the stochastic variable p(t) from its behavior without extrinsic noise (grey line). This shift is mostly determined by $\tau_{z}$ and it is significant only if $\tau_{z}>\tau_{p}$. On the other hand, the shift does not depend on which is the affected parameter (different line-styles) and only weakly depends on the strength of extrinsic fluctuations.

## Data Availability

All data that support the findings of this study are included within the article (and any supplementary files).

## Appendix

### Modelling extrinsic noise

The basic model of gene expression consists of four possible events that occur randomly at exponentially-distributed time intervals, with rates that are constant in the absence of an extrinsic source of noise. The discrete changes in the mRNA and protein populations due to the four events are listed in table A1. The third column shows the event propensity function that determines how often an event occurs.

In the often-valid limit of short-living mRNAs with respect to proteins ($\gamma_{m}\gg \gamma_{p}$), the model can be approximated by a bursty expression model: bursts of protein production arrive at a constant rate $k_{m}$ (as in the Poisson transcription process) and the burst size is given by the number of proteins produced by a single mRNA $b_{p}$. $b_{p}$ is a random variable following a geometric probability distribution with mean burst size $B_{p}=k_{p}/\gamma_{m}$ [88].

**Table A1.** The standard stochastic model of gene expression.

Event	Population reset	Propensity function (f)
mRNA birth	m(t)→m(t)+1	$k_{m}$
mRNA death	m(t)→m(t)−1	$m(t)\gamma_{m}$
Protein birth	p(t)→p(t)+1	$m(t)k_{p}$
Protein death	p(t)→p(t)−1	$p(t)\gamma_{p}$

For the above model, the time derivative of the expected value of any differentiable function φ(m,p) is given by

(A1)
$$\frac{d\langle \varphi (m,p)\rangle }{dt}=\langle \underset{Events}{\sum }\text{Δ}\varphi (m,p)\times f(m,p)\rangle$$

where Δφ(z,m,p) is the change in φ(z,m,p) when an event occurs and f(m,p) is the event propensity function [42]. In particular, the moments of p(t) can be directly obtained from the corresponding Chemical Master equation [89, 90]. For each positive integer n, the time evolution of the expected value of $p(t{)}^{n}$ is given by

(A2)
$$\frac{d\langle p{\left(t\right)}^{n}\rangle }{dt}=\langle G(p)\rangle ,\;n\in \{0,1,2,\ldots \},$$

(A3)
$$G(p):=\sum_{j=0}^{\infty }k_{m}\mathbb{P}\left(b_{p}=j\right)\left[{\left(p+j\right)}^{n}-p^{n}\right]\;\;\;\;+\gamma_{p}p\left[{\left(p-1\right)}^{n}-p^{n}\right].$$

$\mathbb{P}\left(b_{p}=j\right)$ is the probability of having a burst of j protein molecules. From this expression, we can write the equations for the dynamics of the first two moments as

(A4a)
$$\frac{d\langle p\rangle }{dt}=k_{m}B_{p}-\gamma_{p}\langle p\rangle$$

(A4b)
$$\frac{d\langle p^{2}\rangle }{dt}=\gamma_{p}\left(\langle p\rangle -2\langle p^{2}\rangle \right)+k_{m}\langle b_{p}^{2}\rangle +2k_{m}\langle p\rangle B_{p},$$

where we substitute the moments of the burst size geometrical distribution, i.e. $\langle b_{p}^{2}\rangle =2{\langle b_{p}\rangle }^{2}+\langle b_{p}\rangle =2B_{p}^{2}+B_{p}$.

By considering the steady state of equations (A4), the protein mean and noise levels can be calculated as

(A5)
$$p_{ss}=\frac{k_{m}}{\gamma_{p}}B_{p},\;\frac{\langle p^{2}\rangle -{\langle p\rangle }^{2}}{{\langle p\rangle }^{2}}=\frac{1}{p_{ss}}(1+B_{p}):=\eta_{Int,ss}^{2}.$$

The time evolution of the intrinsic noise is:

$$\eta_{Int}^{2}(t)=\frac{1}{\langle p(t)\rangle }\left(1+B_{p}+B_{p}e^{-\gamma_{p}t}\right).$$

This expression, given by equation (2) in the main text, is derived in detail in [50]. Its validity relies on two main assumptions. Firstly, it assumes that the lifetime of mRNA is significantly shorter than that of proteins $\left(\gamma_{m}/\gamma_{p}\gg 1\right)$. Secondly, it requires that the time t is greater than $1/\gamma_{m}$, indicating that enough time must have passed for mRNA levels to reach a steady state.

We now introduce extrinsic noise in the model using an extrinsic factor with copy number z(t) whose stochastic dynamics is modeled as a bursty process with constant production rate $k_{z}$ and degradation rate $\gamma_{z}$. A production event generates a geometrically distributed burst of $b_{z}$ molecules with average burst size $B_{z}$ following

(A6)
$$\mathbb{P}\left\{b_{z}=i\right\}={\left(1-\frac{1}{B_{z}}\right)}^{i-1}\frac{1}{B_{z}},\;i=1,2,3,\ldots$$

The advantage of this phenomenological description is that the extent and the timescale of fluctuations in z(t) can be independently modulated by tuning $k_{z}$, $\gamma_{z}$ and $B_{z}$.

We always consider the cellular factor stochastic process z(t) to be at the steady state characterized by a mean value $z_{ss}$ and ${CV}_{z}^{2}$. For such a process, the steady-state mean $z_{ss}$, the coefficient of variation squared $CV_{z}^{2}$ and the autocorrelation function $R_{z}(\delta t)$ are given by the following relationships:

(A7)
$$z_{ss}=\frac{k_{z}}{\gamma_{z}}B_{z}$$

(A8)
$${CV}_{z}^{2}=\frac{1}{z_{ss}}\left(1+B_{z}\right)$$

(A9)
$$R_{z}(\delta t)=e^{-\gamma_{z}\delta t}.$$

The timescale associated with degradation $\gamma_{z}$ sets the steady-state autocorrelation time of the bursty birth-death process z(t). $1/\gamma_{z}$ describes the average lifetime of a typical fluctuation, as well as the average time separating such fluctuations. Therefore, ${CV}_{z}$ and $\tau_{z}$ respectively represent the extent and the timescale of fluctuations induced in certain parameters of the model.

For any parameter θ of the system, extrinsic fluctuations were implemented by modifying the propensity of the respective reaction such that at any moment the time-dependent rate was $\theta (t)=\theta \frac{z(t)}{\left\langle z\right\rangle }$, with ⟨θ(t)⟩=θ and $CV_{\theta }^{2}=CV_{z}^{2}$.

#### Transcription burst frequency fluctuations

Fluctuations in the extrinsic factor level may impact protein synthesis via its transcription rate, as formalized in table A2. This leads to a system of coupled bursty birth-death processes.

**Table A2.** An effective model of gene expression in the presence of a source of extrinsic noise.

Event	Population reset	Propensity function (f)
Cellular factor birth	z(t)→z(t)+i	$k_{z}\mathbb{P}\left\{b_{z}=i\right\}$
Cellular factor death	z(t)→z(t)−1	$z(t)\gamma_{z}$
mRNA birth	m(t)→m(t)+1	$\frac{z(t)}{z_{ss}}k_{m}$
mRNA death	m(t)→m(t)−1	$m(t)\gamma_{m}$
Protein birth	p(t)→p(t)+1	$m(t)k_{p}$
Protein death	p(t)→p(t)−1	$p(t)\gamma_{p}$

The time-derivative of the expected value of any differentiable function φ(z,m,p) is given by:

(A10)
$$\frac{d\langle \varphi (z,m,p)\rangle }{dt}=\langle \underset{\text{Events}}{\sum }\text{Δ}\varphi (z,m,p)\times f(z,m,p)\rangle .$$

The statistical moments of this joint process evolve as:

(A11)
$$\frac{d\langle p{\left(t\right)}^{n_{1}}z{\left(t\right)}^{n_{2}}\rangle }{dt}=\langle G(p,z)\rangle ,\;n_{1},n_{2}\in \{0,1,2,\ldots \}$$

(A12)
$$G(p,z):=\sum_{j=0}^{\infty }\frac{z(t)k_{m}}{z_{ss}}\mathbb{P}\left(b_{p}=j\right)\left[{\left(p+j\right)}^{n_{1}}z^{n_{2}}-p^{n_{1}}z^{n_{2}}\right]\;\;\;\;\;+\sum_{i=1}^{\infty }k_{z}\mathbb{P}\left(b_{z}=i\right)\left[p^{n_{1}}{\left(z+i\right)}^{n_{2}}-p^{n_{1}}z^{n_{2}}\right]\;\;\;\;\;+\gamma_{p}p\left[{\left(p-1\right)}^{n_{1}}z^{n_{2}}-p^{n_{1}}z^{n_{2}}\right]\;\;\;\;\;+\gamma_{z}z\left[p^{n_{1}}{\left(z-1\right)}^{n_{2}}-p^{n_{1}}z^{n_{2}}\right].$$

[89, 90]. Substituting the appropriate values of $n_{1}$ and $n_{2}$ yields the time evolution of the first and second-order moments of p(t) and z(t)

(A13a)
$$\frac{d\langle z\rangle }{dt}=k_{z}B_{z}-\gamma_{z}\langle z\rangle$$

(A13b)
$$\frac{d\langle p\rangle }{dt}=\frac{\langle z\rangle k_{m}B_{p}}{z_{ss}}-\gamma_{p}\langle p\rangle$$

(A13c)
$$\frac{d\langle z^{2}\rangle }{dt}=k_{z}\langle b_{z}^{2}\rangle +2k_{z}\langle z\rangle B_{z}+\gamma_{z}\left(\langle z\rangle -2\langle z^{2}\rangle \right)$$

(A13d)
$$\frac{d\langle p^{2}\rangle }{dt}=\frac{\langle z\rangle k_{m}\langle b_{p}^{2}\rangle }{z_{ss}}+\frac{2k_{m}\langle pz\rangle B_{p}}{z_{ss}}+\gamma_{p}\left(\langle p\rangle -2\langle p^{2}\rangle \right)$$

(A13e)
$$\frac{d\langle pz\rangle }{dt}=k_{z}B_{z}\langle p\rangle +\frac{k_{m}B_{p}\langle z^{2}\rangle }{z_{ss}}-\gamma_{p}\langle pz\rangle -\gamma_{z}\langle pz\rangle .$$

Solving the above system of differential equations assuming that p(0)=0 and that the extrinsic factor is at steady-state at time t=0 provides both the average p(t) and protein noise level over time:

(A14a)
$$p_{k_{m}}(t)=\frac{\langle z\rangle }{z_{ss}}\langle p(t)\rangle \Rightarrow p_{k_{m}}\left(t_{ss}\right)=\frac{\langle z\rangle }{z_{ss}}\frac{k_{m}B_{p}}{\gamma_{p}}=p_{ss}$$

(A14b)
$$\eta_{k_{m}}^{2}\left(t;{CV}_{z},\tau_{z}\right)=\frac{2e^{\gamma_{p}t}}{{\left(e^{\gamma_{p}t}-1\right)}^{2}}\{\eta_{Int,ss}^{2}\text{sinh}\left(\gamma_{p}t\right)\;\;+\frac{\gamma_{p}^{2}\left[\text{cosh}\left(\gamma_{p}t\right)-\text{cosh}\left(\gamma_{z}t\right)+\text{sinh}\left(\gamma_{z}t\right)\right]-\gamma_{p}\gamma_{z}\text{sinh}\left(\gamma_{p}t\right)}{\gamma_{p}^{2}-\gamma_{z}^{2}}{CV}_{z}^{2}\}.$$

Since $\langle z\rangle =z_{ss}$, the average protein dynamics is not affected by fluctuations of the gene transcription rate.

The steady-state noise levels are now given by:

(A15)
$$\eta_{k_{m},ss}^{2}=\frac{1}{p_{ss}}\left(1+B_{p}\right)+\frac{\gamma_{p}}{\gamma_{p}+\gamma_{z}}{CV}_{z}^{2}\;\;\;\;=\eta_{Int,ss}^{2}+\frac{\gamma_{p}}{\gamma_{p}+\gamma_{z}}{CV}_{z}^{2}.$$

The first component is the intrinsic one while the second component is due to the extrinsic factor contribution [57]. We stress that individual $k_{m}$ or $k_{p}$ fluctuations do not impact the steady-state mean protein levels, given by equation (A5).

#### Translation rate fluctuations

To model noise in the protein translation rate, we modify $k_{p}$ to $k_{p}z(t)/z_{ss}$. This will cause fluctuations of the protein burst size:

(A16a)
$$\langle b_{p}\rangle =\sum_{j=0}^{\infty }\mathbb{P}\left(b_{p}=j\right)j=\frac{z(t)}{z_{ss}}B_{p}$$

(A16b)
$$\langle b_{p}^{2}\rangle =\sum_{j=0}^{\infty }\mathbb{P}\left(b_{p}=j\right)j^{2}=2{\left(\frac{z(t)}{z_{ss}}B_{p}\right)}^{2}+\frac{z(t)}{z_{ss}}B_{p}.$$

The time evolution of moments is then given by:

(A17a)
$$\frac{d\langle z\rangle }{dt}=k_{z}B_{z}-\gamma_{z}\langle z\rangle$$

(A17b)
$$\frac{d\langle p\rangle }{dt}=\frac{k_{m}\langle z\rangle B_{p}}{z_{ss}}-\gamma_{p}\langle p\rangle$$

(A17c)
$$\frac{d\langle z^{2}\rangle }{dt}=k_{z}\langle b_{z}^{2}\rangle +2k_{z}\langle z\rangle B_{z}+\gamma_{z}\left(\langle z\rangle -2\langle z^{2}\rangle \right)$$

(A17d)
$$\frac{d\langle p^{2}\rangle }{dt}=2k_{m}\frac{B_{p}\langle pz\rangle }{z_{ss}}+2k_{m}\frac{B_{p}^{2}\langle z^{2}\rangle }{z_{ss}^{2}}\;\;\;\;+k_{m}B_{p}-2\gamma_{p}\langle p^{2}\rangle +\gamma_{p}\langle p\rangle$$

(A17e)
$$\frac{d\langle pz\rangle }{dt}=k_{z}B_{z}\langle p\rangle +k_{m}\frac{B_{p}\langle z^{2}\rangle }{z_{ss}}-\gamma_{z}\langle pz\rangle -\gamma_{p}\langle pz\rangle .$$

Once again the average protein dynamics is not affected by the extrinsic fluctuations. The steady-state noise levels are now given by:

(A18)
$$\eta_{k_{p},ss}^{2}=\frac{1}{p_{ss}}\left(1+B_{p}\right)+\frac{\gamma_{p}}{\gamma_{p}+\gamma_{z}}{CV}_{z}^{2}+\frac{B_{p}}{p_{ss}}{CV}_{z}^{2}\;\;\;=\eta_{Int,ss}^{2}+\frac{\gamma_{p}}{\gamma_{p}+\gamma_{z}}{CV}_{z}^{2}+\frac{B_{p}}{p_{ss}}{CV}_{z}^{2}\;\;\;=\eta_{k_{m}}^{2}+\frac{B_{p}}{p_{ss}}{CV}_{z}^{2}.$$

The predicted steady-state protein noise in case of protein burst size fluctuations $\eta_{k_{p},ss}^{2}$ exceeds that caused by transcriptions rate fluctuations of $B_{p}CV_{z}^{2}/p_{ss}$. Since in our range of exploration ($B_{p}=5$, $p_{ss}=2000$ and ${CV}_{z}\in [0.1,0.8]$) the contribution due to $B_{p}CV_{z}^{2}/p_{ss}$ is minimal, we assume $\eta_{k_{p}}≃\eta_{k_{m}}$ and we did not explicitly reported the trend $\eta_{k_{p}}(t)$ in the figures of the main text.

#### Protein dilution rate fluctuations

To model noise in the decay rate, we modify $\gamma_{p}$ to $\gamma_{p}z(t)/z_{ss}$. The time evolution of moments is then given by [89, 90]:

(A19a)
$$\frac{d\langle z\rangle }{dt}=k_{z}B_{z}-\gamma_{z}\langle z\rangle ,$$

(A19b)
$$\frac{d\langle p\rangle }{dt}=k_{m}B_{p}-\gamma_{p}\frac{\langle pz\rangle }{z_{ss}},$$

(A19c)
$$\frac{d\langle z^{2}\rangle }{dt}=k_{z}\langle b_{z}^{2}\rangle +2k_{z}\langle z\rangle B_{z}+\gamma_{z}\left(\langle z\rangle -2\langle z^{2}\rangle \right)$$

(A19d)
$$\frac{d\langle p^{2}\rangle }{dt}=k_{m}\langle b_{p}^{2}\rangle +2k_{m}\langle p\rangle B_{p}+\frac{\gamma_{p}\left(\langle pz\rangle -2\langle p^{2}z\rangle \right)}{z_{ss}}$$

(A19e)
$$\frac{d\langle pz\rangle }{dt}=k_{z}B_{z}\langle p\rangle +k_{m}B_{p}\langle z\rangle -\frac{\gamma_{p}\langle pz^{2}\rangle }{z_{ss}}-\gamma_{z}\langle pz\rangle .$$

Note that in this case the moment dynamics is not closed, and the time evolution of lower order moments depends on higher order moments $\left\langle p^{2}z\right\rangle$ and $\left\langle pz^{2}\right\rangle$. To close moment dynamics we use the derivative-matching closure scheme that is consistent with copy numbers following a lognormal distribution [90]. As per this closure, the higher order moments are approximated as

(A20a)
$$\langle pz^{2}\rangle \approx \frac{\langle z^{2}\rangle }{\langle p\rangle }{\left(\frac{\langle pz\rangle }{\langle z\rangle }\right)}^{2}$$

(A20b)
$$\langle p^{2}z\rangle \approx \frac{\langle p^{2}\rangle }{\langle z\rangle }{\left(\frac{\langle pz\rangle }{\langle p\rangle }\right)}^{2}.$$

Substituting the higher order moments in equations (A19) with their corresponding approximation equations (A20) results in a closed system of moment dynamics. Solving these equations results in the steady-state protein number and noise levels:

(A21)
$$p_{\gamma_{p}}\left(t_{ss};{CV}_{z},\tau_{z}\right)\;\;≃\frac{p_{ss}}{2}\left[\sqrt{{\left(1+\frac{\tau_{z}}{\tau_{p}}\right)}^{2}+4\frac{\tau_{z}}{\tau_{p}}{CV}_{z}^{2}}+\left(1-\frac{\tau_{z}}{\tau_{p}}\right)\right]$$

(A22)
$$\eta_{\gamma_{p},ss}^{2}\left({CV}_{z},\tau_{z}\right)\;\;≃\frac{1}{p_{ss}}\left(1+B_{p}\right)+\left\{\frac{1}{2}[\sqrt{{\left(1+\frac{\tau_{z}}{\tau_{p}}\right)}^{2}+4\frac{\tau_{z}}{\tau_{p}}{CV}_{z}^{2}}-\left(1+\frac{\tau_{z}}{\tau_{p}}\right)]\right\}\;\;=\eta_{Int,ss}^{2}+\left\{\eta_{\gamma_{p},Ext,ss}^{2}\right\}$$

when the timescale of the fluctuations matches that of the intrinsic fluctuations of the process p(t), the total variability of proteins number at the steady state is the following:

(A23)
$$\eta_{\gamma_{p},ss}^{2}\left({CV}_{z},\tau_{z}=\tau_{p}\right)≃\eta_{Int,ss}^{2}+\left[\sqrt{1+{CV}_{z}^{2}}-1\right].$$

We use this expression of $\eta_{\gamma_{p},Ext,ss}^{2}=\sqrt{1+{CV}_{z}^{2}}-1$ to estimate the time-evolution of total noise:

(A24)
$$\eta_{\gamma_{p}}(t)≃\sqrt{\eta_{Int}^{2}(t)+{\left(\frac{\gamma_{p}}{p(t)}\frac{\partial \langle p(t)\rangle }{\partial \gamma_{p}}\right)}^{2}\eta_{\gamma_{p},Ext,ss}^{2}}.$$

The results of our simulations mostly agree with our analytical prediction equations (A14) and equations (A24), as we can appreciate in figure A1. However, equations (A24) fails to predict the amplification of the variability added to the system by means of the cellular factor when its typical timescale is longer than that of protein dynamics (in figure A1(B), for long times, the red circles are far above the red continuous line). Additionally, in figure A2(A) we observe a discrepancy between simulations and predictions for the steady state protein level when ${CV}_{z}>0.6$, which further highlights a limitation of our approximated formulas. These quantitative discrepancies show the limitations of the assumption of independence of higher-order moments. Specifically, for high levels of extrinsic noise (high ${CV}_{z}$) or slow fluctuations (low $\gamma_{z}$) the correlations in the dynamics of z and p are more evident and our analytical formulas are less precise.

#### A mathematically controlled comparison

As reported in the main text, extrinsic fluctuations of the protein dilution rate alter the average protein dynamics. The dependence of the final steady-state level of expression on the properties of the source of the extrinsic noise are given by the approximate expression equation (A21). In figure A2 we can appreciate how our estimate predicts the qualitative trends measured during the simulations. In the light of that premise, it is essential to specify the constraints to put different configurations of the model on equal footing for a fair comparison. Basically, to achieve the same steady-state protein level for any choice of the magnitude and the timescale of extrinsic fluctuations, we slightly change the mean values of $k_{m}$, $k_{p}$, $\gamma_{m}$ to reproduce the appropriate $p_{\gamma_{p},ss}\left({CV}_{z},\tau_{z}\right)$, under the constraint that $\gamma_{p},B_{p}$ and $m_{ss}$ are fixed throughout all the different simulation settings.

For any set of values for ${CV}_{z}$ and $\tau_{z}$, the protein expression tends asymptotically to the same equilibrium level independently of the parameter affected by cellular factor fluctuations (figure A3).

### Stochastic simulation specification

Simulations have been implemented using Gillespie’s first reaction algorithm [59]. We simulate the stochastic reactions presented in figure 1 of the main text and in table A1, with the appropriate changes in the propensities due to the cellular factor. Each data point in the figures is the result of $5\times 10^{3}$ trials. As a measure of accuracy, we utilized the bootstrap method to generate a 95% confidence interval for the expression noise. Specifically, we performed $10^{3}$ sampling with repetition from the pools of $5\times 10^{3}$ simulations. This process resulted in a distribution of $10^{3}$ expression noise values. We then calculated the average coefficient of variation and determined the lower and upper error margins as the 2.5th and 97.5th percentiles of these distributions, respectively. Although the error intervals were not depicted in the figures, they were consistently smaller than the marker representing the coefficient of variation.

The standard stochastic model of gene expression allows a discrete number of possible configurations, distinguished by the properties of extrinsic noise $\left({CV}_{z},\tau_{z}\right)$ and the parameter subjected to it.

We analyse the role of extrinsic fluctuations in shaping expression variability; in particular, we explore the functional dependence of η(t) on ${CV}_{z}$, and $\tau_{z}$.

To investigate the role of the extrinsic noise magnitude, we changed the parameter $B_{z}$ and $k_{z}$ to span ${CV}_{z}$ over a certain range, keeping the value of ⟨z⟩ and $\gamma_{z}$ constant so that the timescale of the process z(t) resulted fixed over all the simulations. Conversely, to investigate the role of the timescale of the colored noise, we properly modulated both $\gamma_{z}$ and $k_{z}$ to allow $\tau_{z}$ to span over a certain range while ⟨z⟩ and ${CV}_{z}$ were constant. Moreover, the timescale of the extrinsic noise is always expressed in units of $\tau_{p}$.

We must choose the duration of each simulation. The response times for gene activation and inactivation are both governed by the dilution rate $\tau_{p}=ln(2)/\gamma_{p}$. For proteins that are not actively degraded in the growing cells the response time is equal to one cell generation time [52]. Given this considerations, we set $t_{ss}=20\tau_{p}$ as the interruption time for all the simulations since in the absence of extrinsic fluctuations it would largely exceed the time required for the system to reach the steady state.

In the illustrative examples showed in the main text the parameters have the following values:

(A25)
$$k_{m}=8\;\text{mRNA}\;{\text{min}}^{-1};\;\gamma_{m}=0.2\;{\text{min}}^{-1};\;k_{p}=1\;\text{proteins}\;{\left(\text{mRNA min}\right)}^{-1};\;\gamma_{p}=0.02\;{\text{min}}^{-1}.$$

In this setting, the intrinsic noise is almost negligible because of the low value of the protein burst size $B_{p}=5$ proteins mRNA^−1^. Moreover, at the steady-state $m_{ss}=40\;mRNA$ and $p_{ss}=2000$ proteins. This values represent a relatively highly-expressed gene whose expression variability is mainly due to extrinsic fluctuations [44].

### Protein autocorrelation function in the presence of extrinsic noise

For the standard two-step model of stochastic gene expression, the autocorrelation function for the stochastic variable p(t) is given by

(A26)
$${\text{Φ}}_{p}^{INT}\left(t_{1},t_{2}\right)=\langle p\left(t_{1}\right)p\left(t_{2}\right)\rangle -\langle p\left(t_{1}\right)\rangle \langle p\left(t_{2}\right)\rangle .$$

When the protein level is at the steady state, $\Phi_{p}^{INT}$ is a function of solely the time difference between the two time points considered: $\Phi_{p}^{INT}\left(t_{1},t_{2}\right)=\Phi_{p}^{INT}(t_{1}-t_{2})\;:=\Phi_{p}^{INT}(\delta t)$.

For the bursty standard stochastic model of gene expression the steady state autocorrelation function is approximated by the exponential decay $e^{-(\delta t)(\gamma_{p})}$, which is represented by the grey continuous line of figure A4.

In [64] they investigate a more detailed model of gene expression, explicitly including the dynamics of RNA polymerase and ribosomes as extrinsic noise factors. In our formulation, the same extrinsic noise is captured using the cellular factor z coupled with transcription or translation rates. The protein autocorrelation function $\Phi_{p}(\delta t)$ was reported as the sum of three noise-source specific components, implying a shift of $\Phi_{p}^{INT}(\delta t)$ to higher values [64]:

(A27)
$${\text{Φ}}_{p}(\delta t)={\text{Φ}}_{p}^{EXT,\text{transcription}}(\delta t)\;\;\;\;\;+{\text{Φ}}_{p}^{EXT,\text{translation}}(\delta t)+{\text{Φ}}_{p}^{INT}(\delta t).$$

The relative contribution of these three terms to the total autocorrelation function depends on the typical timescales of the dynamics of RNA polymerase and ribosomes with respect to the timescales of intrinsic fluctuations.

Here, we compare our results on the role of extrinsic noise timescale with this previous analysis in terms of protein autocorrelation time. Indeed, using our modelling framework we also find a shift in the autocorrelation function because of extrinsic parameter fluctuations, which agrees with previous results [64]. This shift depends both on the extent of fluctuations ${CV}_{z}$ (figure A4(A)), as well as on their timescale $\tau_{z}$ (figure A4(B)).

In the limit case of slow extrinsic fluctuations ($\tau_{z}\gg \tau_{p}$, $\gamma_{z}\ll \gamma_{p}$) the extrinsic contribution in equation (A27) dominates the summation. This is consistent with the general idea that in systems with multiple competing timescales, the trend of the autocorrelation function of any chemical species is dominated by the longest autocorrelation time. We can appreciate that in figure A4(B), where the red trends (for $\tau_{z}/\tau_{p}=1000$,) are close to $e^{-(\delta t)(1000\gamma_{p})}$ (lime line).

On the other hand, high-frequency extrinsic fluctuations with $\tau_{z}\ll \tau_{p}$ are filtered out and do not have any impact on the normalized autocorrelation function. In fact, the orange lines are close to the grey line, representing the normalized autocorrelation function for the stochastic variable p(t) in the absence of extrinsic noise.

In conclusion, the sigmoidal extrinsic noise with respect to the timescale of fluctuations shown in figure 3(B) of the main text can also be observed looking at shifts in the protein autocorrelation However, this frequency domain analysis requires the individual trajectories of proteins (p(t)) at single cell level, thus an advanced experimental setup. On the other hand, the results presented in the main text can in principle be drawn solely from time coarse measurements of the average protein number and its variance across a population.

### The effect of extrinsic fluctuations after a reduction of the transcription rate

This section describes the noise dynamics during a shift in expression rate. Specifically, the system is initialized at a steady state with a high protein level characterized by a transcription rate $2k_{m}$. The transcription rate is then halved to $k_{m}$ at a specific time $t^{*}$, and we study the dynamical transition to the lower steady-state protein concentration.

Figure A5(A) shows the dynamics of the average protein number for different choices of the fluctuating parameter. The trend is compatible with the deterministic analytical prediction reported as a grey line in figure A5(A), and given by the expression:

(A28)
$$\langle \hat{p}(t)\rangle =\frac{k_{m}k_{p}}{\gamma_{m}\gamma_{p}}\left(1+\frac{\gamma_{p}e^{-\left(t-t^{*}\right)\gamma_{m}}-\gamma_{m}e^{-\left(t-t^{*}\right)\gamma_{p}}}{\gamma_{p}-\gamma_{m}}\right).$$

After the sudden reduction of the transcription rate, the most frequent cellular reaction is protein degradation, driving the system towards the new steady state. Consequently, extrinsic fluctuations in the protein degradation rate $\gamma_{p}$ have the most significant impact on the overall protein noise, as we can appreciate in figure A5(B).

Once again, to estimate the time evolution of impact of extrinsic fluctuations of any parameter θ of the model, we can evaluate the coefficient $\frac{\theta }{\left\langle \overset{ˆ}{p}(t)\right\rangle }\frac{\partial \langle \overset{ˆ}{p}(t)\rangle }{\partial \theta }$ and fix the initial extrinsic contribution using the measured experimental value $\eta_{Ext}^{2}\left(t^{*}\right)$:

(A29)
$${\hat{\eta }}_{\theta ,Ext}^{2}\left(t;{CV}_{z},\tau_{z}\right)≃{\hat{F}}_{\theta }(t){\hat{G}}_{\theta }\left({CV}_{z},\tau_{z}\right);$$

(A30)
$${\hat{F}}_{\theta }(t)={\left(\frac{\theta }{\langle \hat{p}(t)\rangle }\frac{\partial \langle \hat{p}(t)\rangle }{\partial \theta }\right)}^{2};$$

(A31)
$${\hat{G}}_{\theta }\left({CV}_{z},\tau_{z}\right)={\hat{\eta }}_{\theta ,Ext}^{2}\left(t^{*}\right).$$

These predictions (dashed lines in figure A5(B)) well capture the main trends of the simulation. In conclusion, also in this setting, the noise dynamics clearly distinguish between different dominant sources of extrinsic noise.
